# A Rare Case of Iatrogenic Urinary Bladder Injury During Inguinal Hernia Repair: Management Tips, Tricks and Pitfalls

**DOI:** 10.7759/cureus.63853

**Published:** 2024-07-04

**Authors:** Chrysostomos Kepertis, Maria Tsopozidi, Vassileios Lambropoulos, Sofia Manti, Vassileios Mouravas

**Affiliations:** 1 2nd Department of Pediatric Surgery, Aristotle University of Thessaloniki, Papageorgiou General Hospital, Thessaloniki, GRC

**Keywords:** management tips, children, bladder injury, hernia repair, iatrogenic, rare

## Abstract

Iatrogenic injury of the bladder is a rare incidence during inguinal hernia repair in children, with serious consequences for such patients. Due to the scarcity of information on this matter, it is our goal to share our experience regarding the therapeutic approach to such a rare occurrence. Specifically, a 22-month-old male was admitted to our department with the complaint of vomiting, abdominal pain and anuria, two days after inguinal hernia repair. The child had distention of the inguinal hernia region and was lethargic. The diagnostic investigation did not reveal any significant findings. During surgical exploration, we discovered an injury to the bladder, while a large part of the dome of the bladder was ligated and subsequently became necrotic. After a reoperation and an enduring postoperative course, the patient finally recovered. Currently, the child is under observation. Therefore, it is of paramount importance for pediatric surgeons to be acquainted with the potential for bladder injury during inguinal hernia repair, ways to manage this complication, and various issues that may emerge during the therapeutic process.

## Introduction

Iatrogenic injury to the bladder, during inguinal hernia repair, is a rare occurrence in children, with the potential for serious implications when not promptly recognized. It is a complication more commonly recognized in boys, which is attributed to the higher frequency of inguinal hernia in this population [1]. Herein, we present the case of a 22-month-old boy who suffered a serious injury to the urinary bladder during inguinal hernia repair and elaborate on the applied therapeutic approach.

## Case presentation

**Figure 1:**
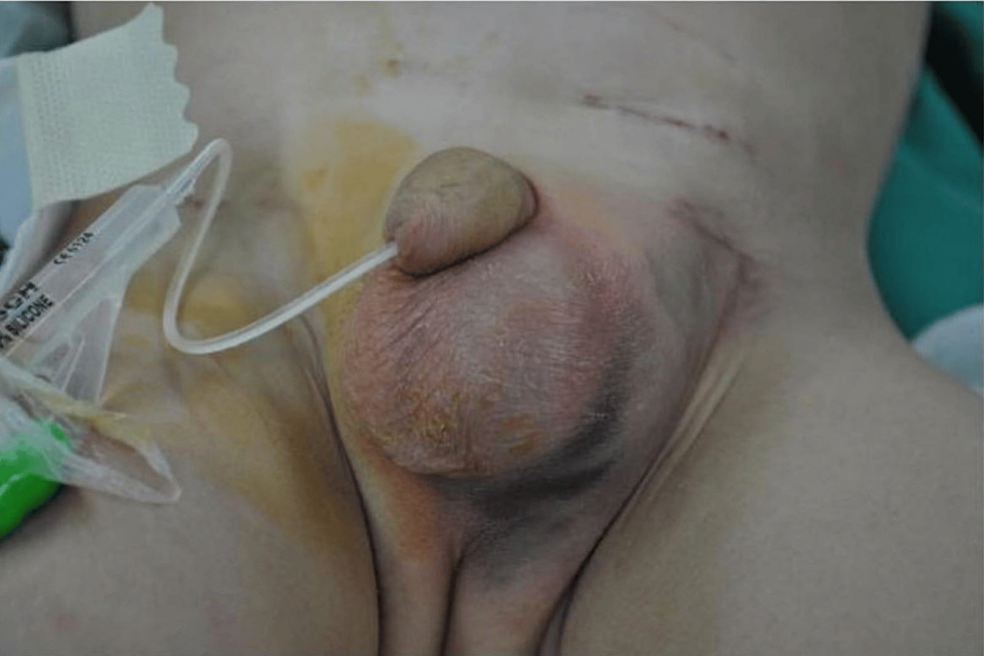
The left inguinal region and scrotum are swollen with bruising

**Figure 2:**
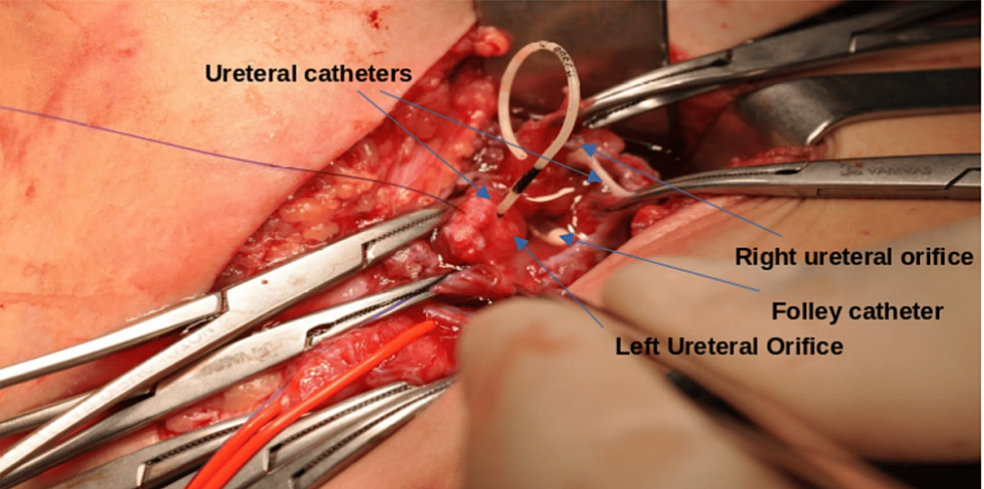
The remaining urinary bladder after resection of the necrotic bladder dome

We report the case of a 22-month-old boy with a history of left inguinal hernia repair two days prior to his hospitalization in our department. The patient presented lethargic with vomiting, abdominal pain, anuria and azotemia with blood urea nitrogen (BUN) of 223 mg/dl and serum creatinine of 4.30 mg/dl. During clinical examination, flatulence and distention of the left scrotal and inguinal regions were observed (Figure 1). Plain films of the abdomen showed intestinal distention and a paucity of colon gas. Following resuscitation and placement of a 10 French (Fr) Foley catheter, the child was transferred to the operating room. The exploration was performed via an expanded previous incision. After cutting the ligature of the presumed hernial sac, a substantial part of the bladder slipped through the inguinal canal. This part was ischemic, deemed non-viable and removed. Placement of 3 Fr Double-J (DJ) stents in both ureters and suturing of the bladder wall followed (Figure 2). A 10 Fr drain was also placed in the retropubic space. The boy was transferred to the Pediatric Intensive Care Unit, where he was hospitalized for four days before returning to the pediatric ward.

Seven days postoperatively the child showed signs of deterioration, presenting once again with symptoms of an acute abdomen, while the Foley catheter was not working properly and he once again was anuric. Efforts to replace the catheter proved futile and the ultrasound (US) showed bilateral ureteral and pelvicalyceal distention, a semi-filled bladder and free fluid collection above the bladder. A second surgery was performed. An 8 Fr Foley catheter was placed this time and the previous incision was reopened. After administering fluids via the catheter, a small leakage was discovered. While opening the peritoneum, clear fluid was bubbling up. A 10 Fr Pezzer catheter was placed in the bladder, as a means of suprapubic urinary diversion. Fibrin sealant was used on the bladder incision and two penrose drains were placed in the retropubic space. Two days postoperatively the child displayed symptoms of deep vein thrombosis of the left lower extremity, which we confirmed ultrasonographically and treated with enoxaparin for several months. After a month of hospitalization and a weary path to recovery, the boy was discharged. Imaging studies performed before his discharge demonstrated bilateral pelvicalyceal dilatation, especially on the left system. The Foley catheter was removed 20 days after the operation, while the Pezzer catheter was closed. It was confirmed that the patient was voiding adequately from the urethra and was subsequently discharged from the hospital.

**Figure 3:**
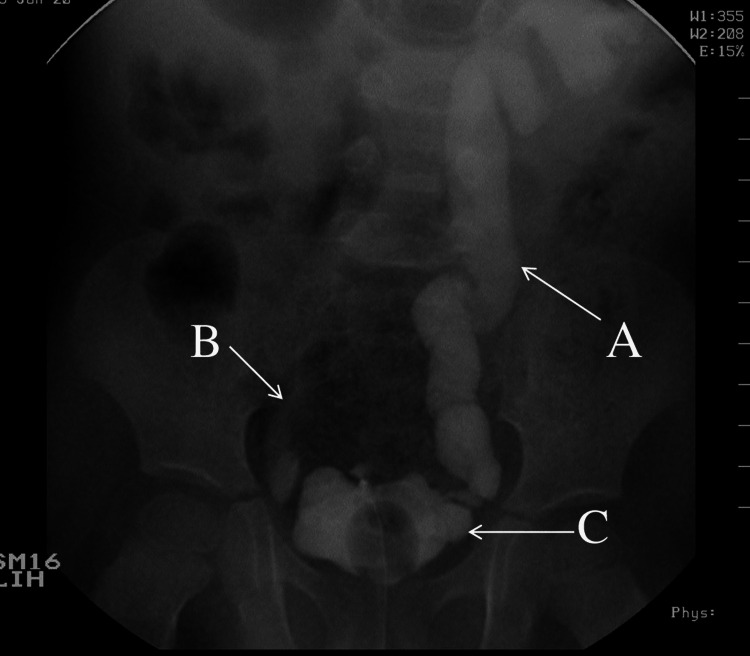
Voiding cystourethrography six months after surgery (A) Grade V vesicoureteral reflux on the left side. (B) Grade I vesicoureteral reflux on the right side. (C) Urinary bladder.

A third surgery was performed a month later, in which the Pezzer catheter and DJs were removed. During cystoscopy, it was observed that the residual bladder was intact and of sufficient capacity. Six months after the surgery, the pelvicalyceal distention had increased and bilateral ureteral distention was observed, both of which worsened during the next six months. At the same time, we performed a retrograde cystography and a MAG3 scan. The former demonstrated bilateral vesicoureteral reflux (VUR) of grade V on the left side and grade I on the right side (Figure 3). The latter demonstrated that the differential function of the right kidney was 48% and the left kidney 52%, with a problematic outflow clearance that markedly improved in the upright position, eliminating the possibility of obstructive uropathy. Moreover, both kidneys had satisfactory function, perfusion, and morphology without signs of scarring. The patient suffered one episode of urinary tract infection (UTI) a year after the surgery, was hospitalized, received intravenous antibiotics and recovered uneventfully. A year after the incident we ordered a urodynamic study, finding a bladder of marginally normal capacity and mildly reduced compliance, without signs of an overactive bladder or urodynamic incontinence.

For the time being, the patient is under continuous prophylactic antibiotics, more specifically trimethoprime/sulfamethoxazole. Moreover, the child is under antispasmodic/anticholinergic (oxybutynin) medication, with an increase in dosage after the urodynamic study. Additionally, in the infection prevention framework and due to the coexistence of phimosis, a circumcision was performed, which was proposed early on, after recovery from the injury, although significantly delayed due to parental refusal. Almost a year and a half after the incidence, he remains asymptomatic, and imaging studies show marked improvement regarding pelvicalyceal/ureteral dilatation and corticomedullary differentiation.

## Discussion

The incidence of intraoperative bladder injury, during herniorrhaphy, in pediatric patients, is estimated at around 0.08%-0.3% and is attributed to bladder ears [1-3]. Bladder ears represent a transient extraperitoneal herniation of the bladder through the deep inguinal ring that occurs in children, usually before six months of age, with an incidence of 9% [1,2,4]. Moreover, they become more prominent when the bladder is partially filled and subside when it is full or at the beginning of micturition [2,4]. Hence they can be misinterpreted as an inguinal hernia [1,5], leading to bladder injury due to ligation of the bladder during closure of the hernial sac [5,6]. According to Duess et al.’s review, 30 cases of bladder injury in children were recognized from 1967 to 2017, 53% of which were accompanied by serious complications and 73% were recognized postoperatively [1]. All serious injuries were diagnosed postoperatively, which confirms the finding that if bladder injuries are promptly recognized, serious complications can be avoided [1,4,6]. If there is suspicion of bladder injury or doubt regarding the nature of the sac, the bladder can be filled with saline intraoperatively [6]. A common practice in our clinic is to urge toilet-trained children to empty their bladders preoperatively or on occasion, we empty the bladder with a Foley catheter in younger children.

Children suffering from serious complications usually present with azotemia, acute abdomen, urinary ascites, anuria, hematuria, ileus, VUR, wound infection, UTIs, and even septic shock [1,5,7]. Hematuria is considered the most common presenting symptom of bladder injury, in up to 95% of cases [7,8]. However, in children with iatrogenic injuries of the bladder, hematuria is not as common and predominantly patients present with anuria, oliguria or an acute abdomen [7]. Most serious injuries present 24 hours to years after the surgery [1]. Likewise, in our case, the child presented 24 hours postoperatively, with an acute abdomen, abdominal distention, anuria, azotemia, hyperkalemia, ileus and urinary ascites. Nevertheless, we did not immediately recognize the injury and misinterpreted the symptoms as a case of recurrent incarcerated inguinal hernia, which is not an uncommon course of events, especially in the event of the first surgery being performed elsewhere [9]. Nonetheless, we immediately proceeded with surgery, so no precious time was lost. Regardless, bladder injury should be considered in the differential diagnosis, especially after inguinal hernia repair. A good diagnostic sign, if the injury is not recognized during surgery, could be voiding difficulties or anuria. We strongly advise physicians not to discharge patients until they have established that the child can urinate adequately after the operation. Generally, if a bladder injury is suspected, the gold standard of diagnosis is CT cystography [8].

Mild injuries can be managed conservatively with suprapubic or transurethral drainage, while significant injuries usually require surgical intervention [1]. Specifically, extraperitoneal and small intraperitoneal injuries, without signs of ileus or sepsis, can be managed solely with a Foley catheter [7,8]. Conversely, intraperitoneal injuries, most of the time, are managed operatively, with a two-layer closure [8]. During the first surgery, we resected the part of the bladder that was established as necrotic, and a two-layer closure was performed on the remainder of the bladder, which basically, included the trigone and a small part of the anterior wall. The injury was immediately above the trigone, sparing the ureterovesical junctions.

Almost a week postoperatively the child once again developed azotemia, abdominal distention and an acute abdomen. The transurethral catheter was obstructed and efforts to replace it were futile. Consequently, the child was reoperated and the clinical deterioration was attributed to leakage, possibly due to high intravesical pressure, caused by an obstructed catheter. Subsequently, we advise, except for a transurethral catheter, the placement of a small-caliber Pezzer catheter suprapubically, to decompress the bladder and avoid complications from an obstructed transurethral catheter. Moreover, we used fibrin sealant to ensure waterproofing of the bladder closure, a practice we consider optional.

In our experience, so far, expectant management is possible if the child shows no sign of renal scarring or deterioration and has adequate voiding patterns. During follow-up our patient suffered from VUR and a single episode of UTI. Despite that, the results of the urodynamic study were promising, since the size of the bladder was deemed adequate for the age of the patient. The patient is managed expectantly, with prophylactic antibiotics, while being observed via urine cultures, ultrasound and urodynamic studies. Specifically, we perform urine cultures every one to two months, an ultrasound every six months and the next urodynamic study is scheduled a year after the first one. In the context of infection prevention and control, we also performed a circumcision. In addition, the patient receives oxybutynin to reduce intravesical pressures and alleviate VUR and subsequent infections. In the same regard, we have to regulate doses carefully; the patient improved after the dosage of the antimuscarinic was increased from 2.5 mg of oxybutynin once daily to 2.5 mg twice daily.

Currently, the patient remains asymptomatic, a year and a half after the incident and nine months after his single so-far UTI incident, with promising results from imaging studies. We believe that in cases of persistent VUR and UTIs, assuming the bladder is of adequate size, endoscopic injection of bulking agents may be an attractive therapeutic modality even before considering bladder augmentation surgery. Furthermore, on the premise of iatrogenic bladder injury prevention, laparoscopic repair of inguinal hernias may be a safe alternative to open surgery. Bladder injuries during laparoscopic surgeries are not uncommon in the adult population; however, they are related to trocar insertion and mesh placement [10]. Laparoscopic herniorrhaphy has the advantage of excellent visualization of organ structures and at the same time, no such incidents have been reported to date, in the pediatric population [6].

## Conclusions

An injury to the bladder may be extremely serious and has major implications. To avoid such dramatic complications, we suggest vigilance even during routine surgeries, such as herniorrhaphy. Management of a child with a major urinary bladder injury is a weary path for both the patient and their family, with a scarcity of information on this matter. Therefore, we hope that this detailed report of the therapeutic process may be of benefit to our colleagues. Moreover, we believe that watchful waiting is a plausible approach, if the child remains fairly asymptomatic, before undertaking any major surgery, such as cystoplasty or ureteral reimplantation.
